# Assessing bias in susceptible–infected–recovered estimation from aggregated epidemic data

**DOI:** 10.1098/rsos.240526

**Published:** 2025-07-23

**Authors:** Naijian Shen, Lydia Bourouiba

**Affiliations:** ^1^ Massachusetts Institute of Technology, Cambridge, MA, USA

**Keywords:** reproduction number, estimation bias, data aggregation, spatial and temporal heterogeneity, sub-epidemics

## Abstract

The canonical susceptible–infected–recovered (SIR) epidemic model is ubiquitous in assessing severity to guide interventions. It is typically applied to hierarchically aggregated data from distinct sub-regions. The introduced heterogeneity can lead to significant errors in estimated epidemic severity. We develop three analytical methods to extract SIR parameters from data, focusing on the reproduction number $R_{0}$ that quantifies epidemic wave severity/strength. The estimation methods are applied to synthetically aggregated incidence data formulated by summing two independent SIR solutions of distinct $R_{0}$ and separated by an onset delay, i.e. temporal offset. When applying the SIR model, we find that $R_{0}$ estimates from the aggregated data can underestimate or overestimate the constituent epidemic waves’ $R_{0}$ even when the prediction appears to agree well with the incidence data, resulting in an erroneous unimodal epidemic dynamics. We find that for two epidemic waves, the stronger the trailing wave, the longer the temporal offset that maintains apparent erroneous unimodal aggregated data. In the special case of two equivalent epidemic strengths, however, the weaker the waves, the longer the offset that maintains apparent unimodal aggregated data. We provide sensitivity analyses with respect to noise perturbation of the data and illustrate our approach using historical influenza data.

## 1. Introduction

Infectious diseases have repeatedly shaped human societies. The COVID-19 pandemic, for example, has led to close to 7 million deaths and over 670 million cases worldwide, as of May 2023 [1]. It adds to a long list of catastrophic epidemics from the plague, 1918 influenza, tuberculosis, Ebola, to HIV–AIDS. Economically, recurring epidemics, like seasonal influenza affecting tens of millions of people every year, are estimated to burden the US economy 87.1 billion dollars per annum [2]. Planning and intervention are therefore critical to mitigate the impact of infectious diseases on society.

Mathematical modelling and data analysis have played central roles in guiding preventative and intervention policies to epidemic outbreaks [3]. Used in conjunction with historical and/or surveillance incidence data, various families and types of data analysis and epidemiological models help quantify a disease’s transmissibility and severity, help assess the future course of spreading and help evaluate potential new routes of intervention and their effectiveness [4–10]. Although progress remains to be done on developing classes of multi-scale mechanistically driven epidemiological models rooted in central detection and/or transmission mechanisms and dynamics [11–14], high-level top-down, relatively compact, models such as the susceptible–infectious–recovered/removed (SIR) ordinary differential equation (ODE) system [15] can help initially assess relative comparisons of scenarios of intervention.

The classical SIR deterministic system, derived from the general stochastic formalism of Kermack & McKendrick [15], is one of the simplest compartmental models that describe the time evolution of an epidemic. An important disease transmissibility parameter identified by the SIR model is the basic reproduction number $R_{0}$, broadly defined as the average number of secondary cases generated by an infected individual [16,17]. A value of $R_{0}>1$ indicates epidemic growth in a population susceptible to infection, whereas a value of $R_{0}<1$ indicates a decline in the spread. $R_{0}$ inferred from surveillance data is a crucial early indicator of the epidemic growth rate routinely used to inform intervention plans. While an analytic solution to the SIR model in integral form exists [18], Schlickeiser & Kröger [19,20] derived an accurate analytic approximate solution explicit in terms of the parameters specifying the SIR model. Based on the approximated solutions, the authors [21] recently developed a monitoring scheme that calculates $R_{0}$ from incidence data, assuming a single epidemic wave. In the present study, we address the potential issues with applying single-SIR theory to aggregated surveillance data obtained through common data collection practices.

Although data collection procedures for infectious diseases vary between countries and governments, a bottom-up hierarchical structure is generally followed: individual incidences are first identified by local healthcare providers before being aggregated and reported to regional and national agencies. Outbreak data are thus repeatedly aggregated. Krishnan *et al*. [22] showed that the multi-regional data collection practices may violate the assumptions of typical epidemiological models that are valid for a single large homogeneous population. Applying the SIR theory to aggregated data can lead to significant errors in predictions. This hierarchical data aggregation increases the degree of complexity of inferring SIR-based disease transmission parameters from observed data. In particular, Krishnan *et al*. [22] found systematic underestimation of $R_{0}$ due to heterogeneity in onset times of sub-regional epidemic waves that were aggregated. Causes of such heterogeneity may include delay and error in data reporting and bias due to geographic and demographic differences. Furthermore, it is expected that if the populations of the sub-regions interact, the epidemics could be affected [23,24]. However, here our focus is on issues of aggregation that are likely to be made for administrative reasons, for example, in a state or country, involving lumping data of regions that are likely not strongly connected, communities in opposite locations of a state or not coupled due to differences in industry/economic structures.

The present theoretical study examines the potential biases associated with analysing aggregated epidemic data using the canonical SIR model. Depending on data availability and modelling constraints, three novel methods of data extraction of the SIR parameters for merged/aggregated surveillance data are formulated. For each method, we compare the resulting estimated SIR model directly against the observational data to assess the model’s applicability. Notably, these methods have potential usage beyond assessing epidemic heterogeneity. We apply the algorithms to synthetically aggregated incidence curves consisting of two isolated underlying epidemics of different dynamics, with a relative delay between their onset times. We present a framework for characterizing the bias and error made by the overall SIR estimation for the merged data in terms of the ground truths. Our analytical approach establishes that significant biases and errors in the estimation of SIR parameters can arise even for ‘perfect’ aggregations in the absence of noise and uncertainty. This reveals the limitations associated with inverting the SIR model in addition to the statistical well-posedness analysed by Melikechi *et al*. [25]. Finally, historical influenza data collected in the US are used to validate our analysis.

## 2. The canonical susceptible–infected–recovered model

Within the SIR framework, infectious disease transmission in a closed population of fixed size $N_{\text{total}}$ is modelled as


(2.1) $$\begin{array}{rl} & \frac{dS}{dt}=-\beta SI,\;\frac{dI}{dt}=\beta SI-\gamma I,\;\frac{dR}{dt}=\gamma I, \end{array}$$

where S(t), I(t) and R(t) are functions of time t. They, respectively, label the population fraction with respect to $N_{\text{total}}$ that is susceptible, infectious and recovered. The infection rate β and recovery rate γ are considered constant. The system’s initial conditions are prescribed by


(2.2) $$\begin{array}{lcr} & S(0)=1-\eta ,\;I(0)=\eta ,\;R(0)=0, & \end{array}$$

where 0<η≪1 is a small parameter. Typically $\eta ∼O(1/N_{\text{total}})$. Equations (2.1) and (2.2) ensure that S+I+R=1 for all t≥0.

During an epidemic outbreak, the incidence rate $\dot{J}$ that reports the newly infected population fraction is given by


(2.3) $$\dot{J}(t)\equiv \beta S(t)I(t)=-\frac{dS}{dt}.$$

Multiplying $\dot{J}$ by $N_{\text{total}}$ gives the rate of raw counts for newly infected cases, which is detection and/or transmission mechanisms and dynamics typically monitored and reported by surveillance agencies as the epidemic curve. Integrating $\dot{J}$ over time produces the total fraction of the population that has ever been infected.

Finally, the basic reproduction number $R_{0}$ is a measure of the average number of cases an infectious individual generates in a wholly susceptible population, and it is defined as


(2.4) $$\begin{array}{lcr} & R_{0}\equiv \frac{1}{k}=\frac{\beta }{\gamma }, & \end{array}$$

where we denote its reciprocal k. We note that the solution space of (2.1) can be completely specified by a vector of parameters $\left(R_{0},\eta ,\beta \right)$. Later, we will show how to construct the inverse mapping from a given SIR solution to its underlying parameters. Note that we list key symbols that we refer to throughout the paper in table 1.

**Table 1.** Table of definitions for key variables introduced for the SIR estimations, in particular in §4, as well as key variables in the synthetic two-wave model that is defined in §3 and analysed in §5.

variables in the SIR estimation methods	
$\dot{J}(t)$	incidence rate as a function of time t
$\dot{J}(0)$	initial incidence rate. Observed for the I-P system (4.10)
$\dot{J}(0)$	initial acceleration of incidence rate. Observed for the SM system (4.9)
${\dot{J}}_{\text{max}}$	peak incidence rate. Observed for the SM system (4.9)
${\dot{J}}_{\text{inf}}$	incidence rate at inflexion. Observed for the I-I system (4.11)
$R_{0}$	the basic reproduction number to be estimated
η	the initial infectious population fraction to be estimated
β	the infection rate to be estimated

variables in the synthetic two-wave data acquisition	
$j_{S}(\tau_{1})$	normalized incidence aggregation as a function of the normalized time $\tau_{1}$ (see (3.3))
$R_{0_{1}},R_{0_{2}}$	variable reproduction numbers of the two sub-waves
$\beta_{1},\beta_{2}$	infection rates of the two sub-waves. By default $\beta_{1}=\beta_{2}=1$
η1,η2	initial infectious fractions of the two sub-waves. By default $\eta_{1}=\eta_{2}=0.0001$
$\bar{\tau }$	rescaled time defined in (3.5) and approximated in (4.8)
${\bar{\tau }}_{d}$	rescaled onset delay that is varied in (3.3) under the normalization (4.8)
${\tilde{j}}_{S}({\bar{\tau }}_{n})$	noisy incidence data as a function of rescaled discrete time ${\bar{\tau }}_{n}$ (see (5.2)).

## 3. Aggregation issues: illustration with two-wave epidemics

To illustrate the issues arising from aggregation of surveillance data, we consider epidemic waves in two closed sub-regions of total population counts $N_{1}$ and $N_{2}$, simulated by the simple SIR equations given in equations (2.1) and (2.2) using parameters $\left(R_{0_{i}},\eta_{i},\beta_{i}\right)$, respectively, for each sub-system. The incidence rates ${\dot{J}}_{i}$ are formed using (2.3) with the following extension:


(3.1) $${\dot{J}}_{i}(t)=\left\{ \begin{array}{ll} \beta_{i}S_{i}(t)I_{i}(t), & \;t\geq 0,\\ 0, & \;t<0, \end{array}\right.$$

where $S_{i}$ and $I_{i}$ are the SIR compartments given by parameters $\left(R_{0_{i}},\eta_{i},\beta_{i}\right)$. Again, recall that our focus is on issues of aggregation that are likely to be made for administrative reasons, for example, in a state or country, and involving lumping data of regions that are likely not strongly connected, communities in opposite locations of a state or not coupled due to differences in industry/economic structures. Thus, we start here by considering two subsystems that are isolated and so with independent constant parameters. Hence, in the above, summing the two sub-regional curves and normalizing with respect to the total population size produces the aggregated incidence rate


(3.2) $$\begin{array}{lcr} & {\dot{J}}_{S}(t;t_{d})=\frac{N_{1}{\dot{J}}_{1}(t)+N_{2}{\dot{J}}_{2}(t-t_{d})}{N_{1}+N_{2}}, & \end{array}$$

where the subscript 1 is assigned to the first wave, ${\dot{J}}_{1}$, whose onset is at t=0, and the subscript 2 denotes the second wave ${\dot{J}}_{2}$, the onset of which is at time $t=t_{d}>0$. For simplicity, we assume in the subsequent analysis that the two sub-regions share the same total population and its initial infectious percentage, i.e. $N_{1}=N_{2}\gg 1$ and $\eta_{1}=\eta_{2}\ll 1$.

We non-dimensionalize (3.2) using the time scale associated with $\beta_{1}$ of the first wave:


(3.3) $$\begin{array}{lcr} & j_{S}(\tau_{1})\equiv \frac{{\dot{J}}_{S}}{\beta_{1}}=\frac{j_{1}(\tau_{1};R_{0_{1}},\eta_{1})+\theta j_{2}(\theta (\tau_{1}-\tau_{d});R_{0_{2}},\eta_{1})}{2}, & \end{array}$$

where


(3.4) $$\begin{array}{lcr} & j_{i}={\dot{J}}_{i}/\beta_{i},\;\tau_{1}=\beta_{1}t,\;\tau_{d}=\beta_{1}t_{d},\;\theta =\beta_{2}/\beta_{1}. & \end{array}$$

Therefore, the evolution behaviour of $j_{S}(\tau_{1};\tau_{d},\theta ,\eta_{1},R_{0_{1}},R_{0_{2}})$ is completely determined by the five independent dimensionless parameters: the delay between onsets of the two SIR waves, $\tau_{d}$; the common initial infectious population, $\eta_{1}$; the reproduction number for each wave, $R_{0_{1}}$ and $R_{0_{2}}$; and the ratio between infection rates, θ. We here define a rescaled non-dimensional time $\bar{\tau }$ using the peak time of the leading wave $\tau_{10}$ as follows:


(3.5) $$\begin{array}{lcr} & \bar{\tau }=\frac{\tau_{1}}{\tau_{10}},\;j_{1}(\tau_{10})=\max_{\tau >0}\;j_{1}(\tau ). & \end{array}$$

We will derive later (in §4.1.3) an approximation for $\tau_{10}$.

**Figure 1.**
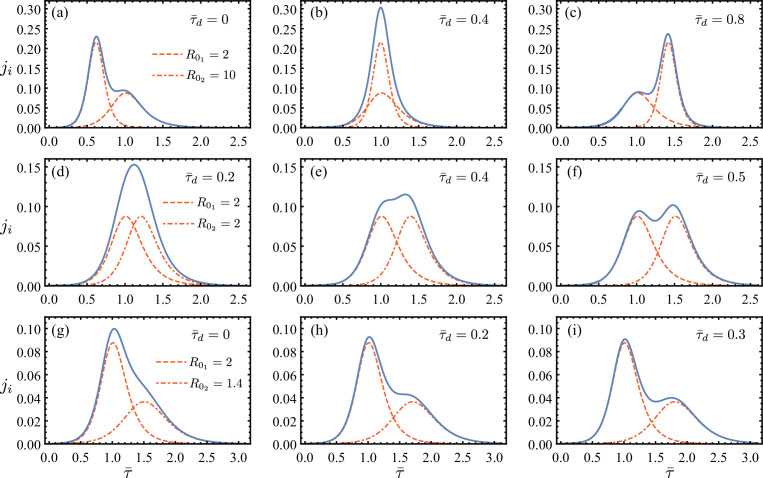
Total incidence rate obtained from summing two relatively delayed epidemic curves of different reproduction numbers. The aggregated rates, $2j_{S}$, are given by the solid lines; the first and second contributing waves, $j_{1,2}$, are plotted using dashed and dot-dashed lines, respectively (see §3 and (3.3) for definitions of $j_{i}$). Effects of increasing delay ${\bar{\tau }}_{d}$ and decreasing $R_{0_{2}}$ are shown. The values of $R_{0_{1}}$ and $R_{0_{2}}$ are given in the first panel of each row. Each row shows the increase in ${\bar{\tau }}_{d}$. Two regimes emerge: (i) a unimodal aggregated regime in which the delay between the two epidemics is small enough, and/or differences in local reproduction numbers are such that the SIR fitted to the aggregated data would lead to an overestimation or underestimation of the reproduction number of the underlying regions (e.g. b,d,g); and (2) a multi-modal aggregated regime, in which the delay between the two epidemics is large enough to reveal the signature of the heterogeneity underlying the aggregated data (e.g. a,c,f,h,i). Statistically, the existence of more than one well-defined local mode in reported incidence counts can be detected for example using the Hartigan's dip test [26]. For the unimodal aggregations here in (b,d,g), each sum is discretized uniformly into 50 data points and fitted using a least-square method to a single-SIR solution [22]. The resulting overall reproduction number for these aggregations in (b,d,g) are $R_{0}=2.44,\;2.08,\;1.18$, respectively.

Figure 1 shows examples of aggregated incidence curves constructed from such two SIR solutions of different reproduction numbers, $R_{0_{1,2}}$, whose onsets are separated by a relative time delay, ${\bar{\tau }}_{d}$, that is rescaled according to (3.5). Note that $2j_{S}$ are given as the sum of two underlying sub-curves. Clearly the combination of $R_{0_{1}}$, $R_{0_{2}}$ and ${\bar{\tau }}_{d}$ leads to the emergence of different regimes: (i) a unimodal aggregated regime, in which the delay between the two epidemics is small enough, and/or differences in local reproduction numbers are such that the SIR fitted to the aggregated data would lead to an overestimation or underestimation of the reproduction number of the underlying regions (e.g. figure 1b,d,g); and (ii) a multi-modal aggregated regime, in which the delay between the two epidemics is large enough to reveal the signature of the heterogeneity underlying the aggregated data (e.g. figure 1a,c,f,h,i).

The potential multi-modal behaviour of $j_{S}$, e.g. in figure 1f, is indeed frequently observed in empirical data for various epidemic outbreaks, as we will show in §6. In such cases, clearly, a simple SIR model does not adequately capture the transmission dynamics. However, one may be misled by the unimodal aggregated regime. If such unimodal regime emerges from the aggregation of data from spatially separated sub-regions with distinct onset times and dynamics, extracting parameters using a simple SIR model introduces important biases and errors.

To illustrate this point more quantitatively, in the next section, we introduce algorithms that extract the SIR parameters to estimate the resulting $R_{0}$ for the aggregated curve in comparison to those of the sub-curves. In particular, we continue to illustrate the issue of aggregation using the example of two independent outbreak waves, with $R_{0_{1}}$, $R_{0_{2}}$ and a delay, ${\bar{\tau }}_{d}$, of the second wave relative to the first. Finally, unless otherwise specified, we use constant values $\eta_{1}=10^{-4}$ and θ=1 throughout and provide sensitivity of our results to the variation of θ in electronic supplementary material, section D(d). Notably, because $j_{S}(\tau_{1})$ constructed in (3.3) is completely parametrized by $\left(\tau_{d},\eta_{1},R_{0_{1}},R_{0_{2}}\right)$ under the mild assumption that the sub-waves share the same initial infections $\eta_{1}=\eta_{2}\ll 1$, effects of different infection rates $\beta_{2}/\beta_{1}$ and recovery rates $\gamma_{2}/\gamma_{1}$ are equivalently covered by simultaneously varying θ and $R_{0_{1,2}}$. We further show in electronic supplementary material, section D(d), that the effects of varying θ can be equivalently reproduced by varying $R_{0_{2}}$ and $\tau_{d}$ for fixed θ=1.

## 4. Susceptible–infected–recovered parameter extraction

It is convenient to continue the normalization introduced in the previous section in the context of the two-region sub-epidemics and to normalize t and $\dot{J}$ using the natural time scale provided by the infection rate β. This gives


(4.1) $$\tau =\beta t,\;j(\tau )=\frac{dJ}{d\tau }=\frac{\dot{J}}{\beta }=S(\tau )I(\tau ),$$

which are both dimensionless. We outline key analytical properties of j(τ) from a single-SIR system for each sub-region next. The derivations of the results are given in electronic supplementary material, section A.

### 4.1. Analytical preliminaries

#### 4.1.1. Initial rate

First, as τ→0, we show that the initial growth of j(τ) is described by the expansion


(4.2) $$\begin{array}{lcr} & j(\tau )=a_{0}+a_{0}a_{1}\tau +O\left(\tau^{2}\right), & \end{array}$$

where $a_{0}=(1-\eta )\eta$ and $a_{1}=1-1/R_{0}-2\eta$ are coefficients parametrized by $R_{0}$ and η.

#### 4.1.2. Maximum rate

We show that a unique maximum of j is attainable, if and only if


(4.3) $$\begin{array}{lcr} & R_{0}>1-2\eta ,\;0<\eta <1/2. & \end{array}$$

The exact peak value is given explicitly by


(4.4) $$\begin{array}{lcr} & j_{\text{max}}=j_{\text{max}}(R_{0},\eta )=(J_{0}-1)(J_{0}-1+1/R_{0}), & \end{array}$$

where $J_{0}=1+W_{-1}\left(\alpha_{0}\right)/(2R_{0})$ is the maximum value of J, with $W_{-1}$ being the Lambert’s W function of order −1 and $\alpha_{0}=2(\eta -1)R_{0}\exp(-R_{0}-1)$.

#### 4.1.3. Peak time

The exact peak time $\tau_{0}$, when $j(\tau_{0})=j_{\text{max}}$ is attained, which we show to be approximated by


(4.5) $$\begin{array}{lcr} & \tau_{*}=\tau_{*}(R_{0},\eta )=\frac{2}{a_{3}}\tanh^{-1}\left(\frac{a_{1}}{a_{3}}\right)\approx \tau_{0}, & \end{array}$$

where $a_{3}=\sqrt{a_{1}^{2}-4a_{0}a_{2}}$ and $a_{2}=[j_{\text{max}}-a_{0}-a_{1}(J_{0}-\eta )]/(J_{0}-\eta {)}^{2}<0$ (see electronic supplementary material, section A).

#### 4.1.4. Inflexion point

Extending Schlickeiser & Kröger [20], we further locate the inflexion point on the rising incidence curve, i.e. $j_{\text{inf}}=j(\tau_{\text{inf}})$ that satisfies $j\prime \prime (\tau_{\text{inf}})∼0$. The inflexion point is often of interest for epidemic intervention. The explicit expression follows:


(4.6a)jinf=jinf(R0,η)=−a326a2,(4.6b)τinf=τinf(R0,η)=τ∗−cosh−1⁡(2)a3,

provided that $a_{1}/a_{3}>1/\sqrt{3}$.

#### 4.1.5. Final value

As τ→∞, we further show that the total population that had been infected is given by


(4.7) $$J_{\infty }(k,\eta )=\int_{0}^{\infty }\dot{J}(t)dt=1+\frac{1}{R_{0}}W_{0}\left((\eta -1)R_{0}e^{-R_{0}}\right),$$

where $W_{0}$ is the principal Lambert’s function.

#### 4.1.6. Domain of unimodality, D

If the delay between two epidemics is large enough, the heterogeneity underlying the aggregated data is revealed by the existence of more than one well-defined local mode (e.g. see figure 1a,c,f,h,i). Statistically, the existence of multi-modal patterns in reported incidence counts can be detected, for example, using the Hartigan’s dip test [26]. We focus on the cases where a single mode emerges from these aggregations, but would still lead to major error in the assessment of the parameters because the regions aggregated are inherently highly distinct in their local epidemic dynamics. To quantify this effect, we here define the domain of parameters for which a unimodal aggregated curve would emerge.

We denote by D the parameter space given by $R_{0_{1}}$, $R_{0_{2}}$ and ${\bar{\tau }}_{d}$ that yields a single peak in $j_{S}$ as a function of $\bar{\tau }$. Here, the evolution time $\bar{\tau }$ as well as the onset delay ${\bar{\tau }}_{d}$ are again measured against the peak time of the first wave, using rescaled non-dimensional time


(4.8) $$\begin{array}{lcr} & \bar{\tau }=\frac{\tau_{1}}{\tau_{*}(R_{0_{1}},\eta_{1})}, & \end{array}$$

where $\tau_{*}$ is now the approximation to $\tau_{0}$ given in (4.5).

**Figure 2.**
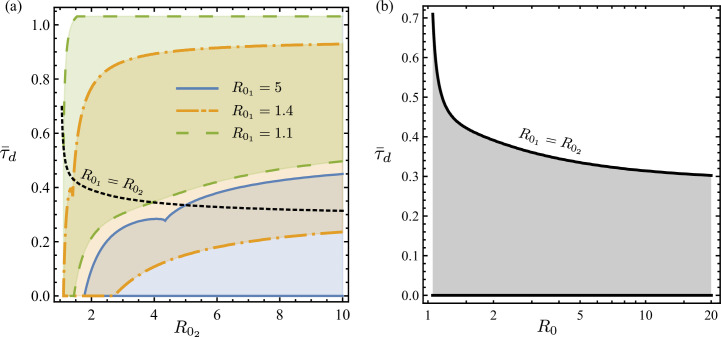
(a) Regions of unimodal $j_{S}(\bar{\tau };{\bar{\tau }}_{d}$, $R_{0_{1}}$, $R_{0_{2}}$ as a function of $\bar{\tau }$ in the parameter space of $R_{0_{1}}$, $R_{0_{2}}$ and ${\bar{\tau }}_{d}$, where ${\bar{\tau }}_{d}$ is the rescaled delay time according to (4.8). The region of $(R_{0_{2}}$,${\bar{\tau }}_{d})$ bounded by the solid lines is shown for fixed $R_{0_{1}}=5$, and similarly dot-dashed lines are used for $R_{0_{1}}=1.4$, as well as dashed lines for $R_{0_{1}}=1.1$. The maximum ${\bar{\tau }}_{d}$ that allows unimodality for variable $R_{0_{1}}=R_{0_{2}}$ is given by the dotted line. (b) The special case of permissible delay ${\bar{\tau }}_{d}$ between two identical sub-epidemics of reproduction $R_{0_{1}}=R_{0_{2}}$ that produce a unimodal aggregated epidemic. Refer to §4.1.6 for details. Note that for a given $R_{0_{1}}$, the maximum delay ${\bar{\tau }}_{d}$ between the first and second waves that continues to enable a unimodal aggregation, i.e. defines the upper boundary of D, increases with $R_{0_{2}}$. In other words, for a given initial epidemic wave, the stronger the trailing wave, the longer the offset that continues to ensure apparent unimodal aggregated data. However, in the special case of $R_{0_{2}}=R_{0_{1}}$, the weaker the common waves, the longer the offset that can continue to ensure apparent unimodal aggregated data.

We solve for the domain, D, for which $dj_{S}/d\tau_{1}=0$ has a unique solution for $\tau_{1}$, using (3.3). Figure 2 shows the boundaries of three such unimodal regions in terms of $R_{0_{2}}$ and ${\bar{\tau }}_{d}$ obtained for constant $R_{0_{1}}=5$, 1.4, 1.1, as well as variable $R_{0_{2}}=R_{0_{1}}$. Note that for each $R_{0_{1}}$ in figure 2a, the maximum ${\bar{\tau }}_{d}$ that defines the upper boundary of D overall increases with $R_{0_{2}}$, suggesting that a unimodal aggregation can emerge with longer delays between sub-curves if the first wave is followed by a strong second wave, i.e. a second wave with much higher $R_{0}$ than the first wave (e.g. see the first row of figure 1).

Figure 2b shows the boundary line, below which unimodal solutions of the SIR emerge, i.e. D exists in a ${\bar{\tau }}_{d}$ as a function of $R_{0}$ representation. This plot shows the special line of $R_{0_{2}}=R_{0_{1}}$ in particular. That line decreases as $R_{0}$ increases. This implies that longer delays can enable unimodality for two identical weak epidemic curves. For example, delays as large ${\bar{\tau }}_{d}=0.5$ or 0.45 still enable unimodality for $R_{0}=1.17$ and 1.29, respectively.

### 4.2. Parameter extraction

Having established exact and explicit expressions for j(τ) at τ=0 (4.2) and $\tau =\tau_{0}$ (4.4), as well as approximants $\tau_{*}$, $\tau_{\text{inf}}$ and $j_{\text{inf}}$, it is possible to construct systems of three independent equations, leading to solutions that map the generally observable data $\dot{J}(t)$ to a unique set of SIR parameters $\left(R_{0},\eta ,\beta \right)$. The resulting parameters reproduce desirable features of $\dot{J}$ in a single-SIR wave. We formulate these three distinct algebraic systems in this section. Depending on different data availability at various stages of an epidemic and potentially specific modelling constraints, one or more of these methods can be employed in isolation or jointly in practice.

Using the resulting parameters from each system to integrate the SIR equations leads to a corresponding SIR model for $\dot{J}(t)$ that is generally different among the three methods. Compared to the common optimization approach of fitting observational data to an SIR solution, by design the SIR model produced by the present methods is faithful to specific input data. Hence, the resulting discrepancy between the SIR estimate and data not built into the solution can be used for direct assessments of the single-wave SIR theory.

#### 4.2.1. The shooting method

First, we formulate the following system obtained from combining (4.2) and (4.4):


(4.9) $$\beta a_{0}=\dot{J}(0),\;\beta^{2}a_{0}a_{1}=\ddot{J}(0),\;\beta j_{\text{max}}={\dot{J}}_{\text{max}},$$

where $a_{0}$, $a_{1}$ and $j_{\text{max}}$, respectively, given in (4.2) and (4.4), depend on η and $R_{0}$ only, whereas the initial incidence rate $\dot{J}(0)$, its initial acceleration $\ddot{J}(0)=(d\dot{J}/dt){|}_{t=0}$ and peak value ${\dot{J}}_{\text{max}}$ are considered as measurements from the reported incidence curve. The algebraic system (4.9) is numerically solvable under most realistic conditions of $\dot{J}$ to produce solutions that satisfy 1/2>η>0, $1/(1-2\eta )<R_{0}$, β>0. We prove the existence and uniqueness of such solutions in electronic supplementary material*,* section B. For η≪1, we solve (4.9) asymptotically in the $R_{0}\to \infty$ and $R_{0}\to 1/(1-2\eta )$ limits in electronic supplementary material, section C(a), and compare with numerical solution in electronic supplementary material, figure S1.

#### 4.2.2. The two-point methods

Other algebraic systems of equations similar to (4.9) can be formed to take advantage of different properties of the analytic approximant j. Here, we utilize two pairs of points on a given incidence curve to infer its underlying SIR parameters: first, the initial and the peak point, henceforth referred to as the I-P system, and second, the initial and the inflexion point, abbreviated as the I-I system.

##### The I-P system

By prescribing the analytic j approximant initial value at τ=0 and peak value at $\tau =\tau_{*}$, one has


(4.10) $$\begin{array}{lcr} & \beta a_{0}=\dot{J}(0),\;\beta j_{\text{max}}={\dot{J}}_{\text{max}},\;\frac{\tau_{*}}{\beta }=t_{\text{max}}, & \end{array}$$

where $a_{0}$, $j_{\text{max}}$ and $\tau_{*}$ are given in equations (4.2), (4.4) and (4.5), respectively; $\dot{J}(0)$ is the initial incidence rate from observed data, $t_{\text{max}}$ is the peak time of maximum incidence rate ${\dot{J}}_{\text{max}}$. Again, (4.10) is closed for parameters $\left(R_{0},\eta ,\beta \right)$.

##### The I-I system

Similarly, replacing the boundary condition in (4.10) at the peak point with one at the inflexion point leads to


(4.11) $$\begin{array}{lcr} & \beta a_{0}=\dot{J}(0),\;\beta j_{\text{inf}}={\dot{J}}_{\text{inf}},\;\frac{\tau_{i}}{\beta }=t_{\text{inf}}, & \end{array}$$

where $j_{\text{inf}}$ is found in (4.6*a*) and $\tau_{\text{inf}}$ in (4.6*b*); ${\dot{J}}_{\text{inf}}$ and $t_{\text{inf}}$, if they exist, are, respectively, the incidence rate value and the time of its occurrence measured at the first inflexion point of the rising $\dot{J}$.

We derive the conditions that $\dot{J}$ data must satisfy for a unique solution of the SIR parameters to exist for both I-P and I-I systems in electronic supplementary material*,* equations (B.14) and (B.15), respectively. We provide the numerical solutions of k that follow from (4.10) and (4.11) in electronic supplementary material, table S1 and figure S2.

### 4.3. Method comparison

We remark on the key differences between the three proposed methods, in terms of their capabilities inferring SIR parameters from a given epidemic incidence curve. First, if the observed $\dot{J}$ data follow exactly from the solutions of an SIR system, then under the mild condition given in electronic supplementary material*,* equation (B.9), that particularly ensures solution uniqueness for the shooting method (SM), all three proposed methods yield the same SIR parameters that recover the SIR solution of the observed data.

However, if for any method the $\dot{J}$ measurements fail the solution existence test (electronic supplementary material*,* equations (B.14) and (B.15)), it follows that a simple SIR model cannot capture the data. This existence test differentiates the present methods from the fitting approach where a best-fit solution almost always exists and assessing model suitability is generally difficult.

More practically, when using the SM, when a solution exists but the observed incidence rate cannot be perfectly captured at all times by a simple SIR model, the SIR estimate for the data, by design, is faithful to the input initial value and derivative, as well as the peak amplitude of the observed $\dot{J}(t)$ curve. Since the peak time is not built into the SM formulation, a different trajectory compared to the data is expected from the calculated SIR estimate, reaching maximum $\dot{J}$ at a time that possibly deviates from the observed value.

In contrast, parameters obtained using the two-point methods produce an SIR solution that, again by design, passes through two points of choice on the incidence curve, namely, $\left(\dot{J}(0),{\dot{J}}_{\text{max}}\right)$ for the I-P system (4.10), and $\left(\dot{J}(0),{\dot{J}}_{\text{inf}}\right)$ for the I-I system (4.11). Again, in both cases, a different trajectory connecting the chosen points is permissible by the estimates compared to the observed data. Furthermore, if the inflexion point can be reasonably identified on the incidence curve, the I-I method can also be applied before the peak is observed, giving a unique estimate for the peak time and amplitude.

Although all three methods depend heavily on the quality of the empirical data, the two-point methods inherit stronger tolerance against input noise and uncertainty, as we discuss in electronic supplementary material*,* section C.

## 5. $R_{0}$ estimation

In this section, we apply the three methods developed previously for SIR parameter extraction, namely the shooting, the two-point I-P and the two-point I-I methods, to the synthetic incidence curve $j_{S}$, defined in (3.3) from combining two independent epidemic waves. The operations are restricted to the solvable subsets of the unimodal domain D, leading to solutions for the SIR parameters $\left(R_{0_{\mu }},\eta_{\mu },\beta_{\mu }\right)$ for each method. Here, μ∈{SM,I-P,I-I} denotes the abbreviated method name. Additionally, the complement sets in D where a particular method fails are also excluded because of the inability of the simple SIR theory to capture the corresponding desired data feature. Specifically, the required observations in this case, including the initial incidence rate $\ddot{J}(0)$ for μ=SM and the incidence inflexion point $\dot{J}(t_{\text{inf}})={\dot{J}}_{\text{inf}}$ for μ=I-I can be directly calculated from the construction


(5.1) $$\begin{array}{lcr} & \dot{J}={\dot{J}}_{S}=\beta_{1}j_{S}(\tau_{1}), & \end{array}$$

where $j_{S}$ is given by (3.3), and $\beta_{1}$ is arbitrary if normalized time $\tau_{1}$ is used. Therefore, we select $\beta_{1}=1$ without loss of generality for computation. We further explain the formulation and solution processes in electronic supplementary material*,* section D(a), and discuss the solvable subsets of D in electronic supplementary material*,* section D(b). Finally, we use each resulting parameter vector to integrate equations (2.1) and (2.2), generating the corresponding SIR estimate $j_{\mu }={\dot{J}}_{\mu }/\beta_{1}$ for the aggregated two-wave data.

As a reminder, we list the key symbols that we refer to in the following result sections in table 1. This includes variables denoted in §4 for the SIR estimation methods, as well as variables used in the construction of the two-wave data.

### 5.1. Typical results

**Figure 3.**
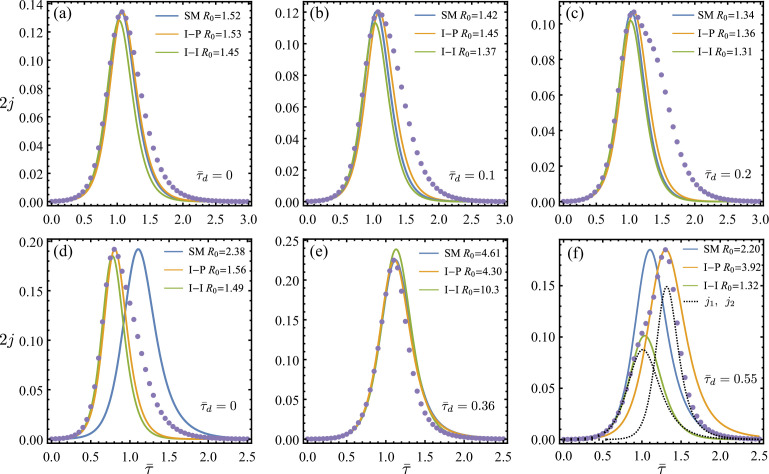
SIR estimation for the synthetic two-wave sum $j_{S}$ (circles) obtained using three approaches: the SM (blue lines), the I-P method (orange lines) and the I-I method (green lines). The aggregated $j_{S}$ are constructed using $R_{0_{2}}=1.66$ in (a–c) and $R_{0_{2}}=3$ in (d–f), with increasing delay of the second wave ${\bar{\tau }}_{d}$ relative to a common first wave of $R_{0_{1}}=2$. The underlying two sub-waves are particularly given in (f) by the dotted lines to show their contributions to $j_{S}$. The $R_{0}$ estimates associated with different methods are compared. Overall, we observe that significant error between the aggregated SIR models, and the underlying epidemics can occur in the decay stage, even though the rising stage of the epidemics is well captured.

We present in figure 3 two sets of sequential results as ${\bar{\tau }}_{d}$ increases for $R_{0_{2}}=1.67$ and $R_{0_{2}}=3.33$, with a common $R_{0_{1}}=2$. The SIR reconstructions $j_{\mu }$ obtained using all three estimation methods are compared against the original aggregated data $j_{S}$.

In the first example, given in figure 3a–c, where $R_{0_{2}}=1.7<R_{0_{1}}$, all methods perform similarly to each other in the range of delays used. These estimates successfully capture the rising part of $j_{S}$ but predict a significantly faster decay than the synthetic data. Such discrepancy is amplified as ${\bar{\tau }}_{d}$ increases, and the $j_{2}$ component of $j_{S}$ starts to manifest at large times. All three methods consistently give overall $R_{0}$ estimates that are smaller than both of the ground truths, $R_{0_{1}}$ and $R_{0_{2}}$. In this case, the severity of the outbreak is underestimated in terms of both incidence prediction and reproduction number.

Differences between methods become evident in the second case shown in figure 3d–f, where $R_{0_{2}}=3.3>R_{0_{1}}$. At small delay ${\bar{\tau }}_{d}=0^{+}$, the SM generates a peak time significantly later than the observation for $j_{S}$, while the other two methods show excellent agreement with data nearly up to the observed peak. Nonetheless, after the peak time, decaying rates of $j_{\mu }$ given by both I-P and I-I methods are markedly larger than the data. As ${\bar{\tau }}_{d}$ increases in figure 3e, the three methods show similar results with improving performance explaining the synthetic data.

It is therefore possible to determine an optimal delay ${\bar{\tau }}_{d}^{*}$ for each method that minimizes the distance between $j_{\mu }$ and $j_{S}$ so that the reconstructed SIR estimates agree reasonably well with the synthetic curve for all time. This is discussed further shortly.

Increasing ${\bar{\tau }}_{d}$ can cause $j_{\text{SM}}$ to peak ahead of $j_{S}$ (see figure 3f). Importantly, the $j_{2}$ contribution in this case (figure 3f) takes over the rising part of $j_{S}$ before its peak. This must be captured by the I-I method that conforms to the first inflexion point on the synthetic curve. However, the I-P method here is by design not able to capture the emerging curvature distortion occurring as $j_{S}$ rises, and the decaying rate of $j_{\text{I-P}}$ is consistently slower than the data.

We observe that our SIR estimation methods yield incidence curves in good agreement with the aggregated data up to the peak point for the SM and I-P method and up to the inflexion point for the I-I method, but fail to extrapolate satisfactorily beyond these limits. This illustrates the limitation of blindly applying an SIR model to aggregated epidemic data. In general, none of the three methods reconstructs perfectly a simple SIR model $j_{\mu }$ that exactly recovers the synthetic aggregation $j_{S}(\bar{\tau };{\bar{\tau }}_{d},R_{0_{1}},R_{0_{2}})$ as a function of $\bar{\tau }$ for arbitrary parameters in D.

### 5.2. Noise responses

Here, we theoretically demonstrate the sensitivity of our SIR estimation methods responding to random noise. This is achieved by first discretizing the synthetic two-wave incidence curve $j_{S}$ over a uniform grid $\left\{{\bar{\tau }}_{n}=3n/N|n=0,1,...,N\right\}$, where ${\bar{\tau }}_{n}$ is the nth normalized time point given by (4.8) and N is the grid size. To mitigate undesired numerical errors associated with discretization in this study of noise sensitivity, we choose a sufficiently large N∼O(100). This ensures that all observables of interest, including the initial growth rate ${\ddot{J}}_{0}$, the inflexion point $\left(t_{\text{inf}},{\dot{J}}_{\text{inf}}\right)$, and the peak point $\left(t_{\text{max}},{\dot{J}}_{\text{max}}\right)$ of (5.1) can be approximated with more than 99% accuracy using discrete $j_{S}(\tau_{*}\;{\bar{\tau }}_{n})$ (given by (3.3)) and its time derivatives (given by a fourth-order finite difference scheme). We then introduce random noise to the discrete values $j_{S}(\tau_{*}{\bar{\tau }}_{n})$ for all n using the normal distribution N as follows:


(5.2) $$\begin{array}{lcr} & {\tilde{j}}_{S}({\bar{\tau }}_{n})=j_{S}(\tau_{*}\;{\bar{\tau }}_{n})+N\left(0,\sigma \;\max_{0\leq n\leq N}\;j_{S}(\tau_{*}\;{\bar{\tau }}_{n})\right), & \end{array}$$

where the noise level 0<σ<1 defines a portion of the maximum $j_{S}$ for s.d.

To apply our SM, I-P and I-I methods of SIR estimation for the noisy data ${\tilde{j}}_{S}$, the required incidence statistics are calculated next. Specifically, we estimate the inflexion point, the peak point and the initial growth rate by least-square fitting three data subsets to a fourth-order polynomial, a second-order polynomial and an exponential function, respectively. This process is explained in detail in electronic supplementary material, section D(e).

**Figure 4.**
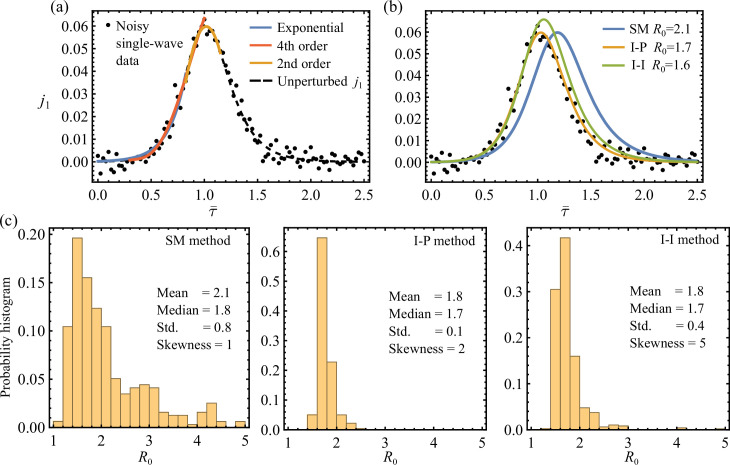
(a) Perturbed discrete single-SIR wave ${\tilde{j}}_{S}={\tilde{j}}_{1}$ with 5% Gaussian noise. The dashed line shows the underlying continuous $j_{1}$ of $R_{0}=1.67$. The solid lines show the different best-fits over subsets of data used to determine the initial growth rate, the inflexion point and the peak point. (b) The SIR estimations for the noisy data given by the SM, I-P and I-I methods (see the blue, orange and green lines, respectively). The corresponding $R_{0}$ estimates are labelled. (c) Histograms and statistics of the $R_{0}$ estimates obtained by three different methods over one hundred ${\tilde{j}}_{1}$ datasets of the same parameters (*σ* = 0.05, *R*_0_ = 1.67). We show that all three methods generate most probable $R_{0}$ estimates that are close to the underpinning value of $R_{0}=1.67$. Overall the I-P method has the best accuracy and consistency, whereas the SM performs the worst.

First as a baseline comparison, results obtained for a noisy single-SIR wave are shown in figure 4. Here, the incidence data were simulated using σ=5% in (5.2) and $R_{0_{1}}=R_{0_{2}}=1.67$, ${\bar{\tau }}_{d}=0$ in (3.3), such that the two-wave model collapses to a single-SIR solution. Specifically, the best-fit functions used to calculate the initial, inflexion and peak statistics from a typical noisy epidemic curve are marked in figure 4a. In this case, all three SM, I-P and I-I algebraic systems are solvable, leading to their corresponding SIR reconstructions and the associated $R_{0}$ estimates given in figure 4b. We further sampled (5.2) independently 100 times using the same parameters ($\sigma =5\%,R_{0}=1.67$) and then applied the three SIR estimation methods to these noisy single-SIR datasets. For each method, a probabilistic histogram of the resulting $R_{0}$ estimates is given in figure 4c. We verify that all three methods produce most probable $R_{0}$ estimates that are close to the underlying unperturbed value of $R_{0}=1.67$. The I-P method particularly has the best accuracy and consistency at recovering $R_{0}$ by demanding both initial and peak points at their realized times. In contrast, the SM captures the observed peak magnitude without constraining its time of occurrence. Therefore, the strong sensitivity of the modelled peak time of SM with respect to initial conditions, as discussed in §4.3 and electronic supplementary material, section C, allows significantly larger error in parameter estimation. And here the relative noise near initial times is particularly large, as seen in figure 4a. Indeed, the $R_{0}$ distribution given by the SM in figure 4c suggests that excessively overestimated $R_{0}$ can arise when the predicted peak time does not match the data. An example of moderately delayed peak is shown in figure 4b. In fact, both premature and delayed peak time predictions can lead to severe $R_{0}$ overestimation. This can be demonstrated using the dimensionless peak time $\tau_{*}(R_{0},\eta )$ given in (4.5), with $R_{0}$ and η substituted by the asymptotic solutions of the SM for large $R_{0}$ (see equation (C.6) in electronic supplementary material, section C). The resulting $\tau_{*}$ is not a monotonic function of $R_{0}$ when η is fixed, implying that an increased $R_{0}\gg 1$ can cause $\tau_{*}$ to move in both directions. This in turn explains the larger spread of the $R_{0}$ estimates of the SM.

**Figure 5.**
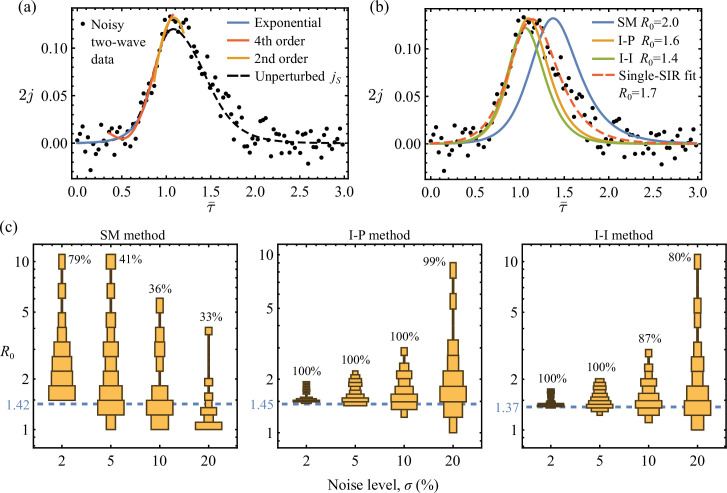
(a) Perturbed discrete two-wave sum ${\tilde{j}}_{S}$ with 10% Gaussian noise in (5.2). The dashed line shows the underlying continuous $j_{S}$ that is identical to figure 3b, and the solid lines show the different best-fits over subsets of data used to determine the initial growth rate, the inflexion point and the peak point. (b) The SIR estimations for the noisy data given by the SM, I-P and I-I methods (see the blue, orange and green lines, respectively), as well as the nonlinear fit according to the single-SIR equations (dashed line with best-fit parameters β=0.89, η=0.00028, $R_{0}=1.72$). All corresponding $R_{0}$ estimates are labelled. (c) Histograms of the $R_{0}$ estimates obtained by three different methods over one hundred ${\tilde{j}}_{S}$ datasets for each noise level *σ* = 0.02, 0.05, 0.1, 0.2. For each method, the dashed line gives the reference $R_{0}$ prediction obtained for the unperturbed $j_{S}$ (see also figure 3b). And for each σ, the method's solvable rate $p_{\mu }$ is labelled in percentage. We demonstrate again that the I-P and I-I methods are robust against noise, consistently and most probably generating $R_{0}$ estimates that are close to the unperturbed prediction values.

Next, we show analogous results obtained for discrete two-wave data. Figure 5a gives a perturbed dataset ${\tilde{j}}_{S}$ of noise level of σ=0.1 based on the continuous two-wave sum $j_{S}$ of $R_{0_{1}}=2$, $R_{0_{2}}=1.67$ and ${\bar{\tau }}_{d}=0.1$. The same continuous $j_{S}$ is previously plotted in figure 3b. Again, the best-fit functions are also shown in figure 5a. In this case, applying the SM, I-P and I-I methods leads to their corresponding SIR reconstructions given in figure 5b. Compared to the $R_{0}$ estimates of figure 3b that are obtained for the unperturbed two-wave model $j_{S}$, we find that the $R_{0}$ estimates obtained here with 10% noise by the SM, I-P and I-I methods are 40, 10 and 2% higher, respectively. Nonetheless, the SIR curves following the I-P and I-I methods match the noisy data convincingly. Additionally, in figure 5b, we further compare the incidence curve obtained by least-square fitting the discrete data to the numerical solution of the single-SIR (2.1). The fitted value of $R_{0}=1.7$ here is comparable to the other estimation results. Notably, although the fitted SIR curve matches the data better overall, it appears nearly identical to the I-P and I-I curves up to peak epidemic. This observation has significant implications for live epidemic surveillance that we will further discuss in §7.

At each noise level, ranging from *σ* = 2, 5, 10, 20%, we obtained 100 datasets from sampling (5.2) and applied the three SIR estimation methods to each of them. Notably, for a given noisy dataset, application of a particular method is considered solvable only if it produces an SIR reconstruction. Unsolvable cases can arise if the required statistics that we observed by fitting fall outside the method’s solvable domain (see electronic supplementary material, section D(a)). The percentage of solvable applications, $p_{\mu }(\sigma )$, for each method μ∈{SM,I-P,I-I} as a function noise level σ measures the method’s robustness against noise. Among the solvable applications, the distribution of the resulting $R_{0}$ estimates associated with each method is given as function of σ in figure 5c, where $p_{\mu }$ is also labelled for each σ. Similar $R_{0}$ distributions are formed by the I-P and I-I methods at all noise levels, with their most probable $R_{0}$ predictions located close to the reference values ($R_{0_{\text{I-P}}}=1.45$ and $R_{0_{\text{I-I}}}=1.37$) obtained in figure 3b for the unperturbed continuous $j_{S}$. For both I-P and I-I methods, the probability of finding $R_{0}>R_{0_{\text{I-P,I-I}}}$ is higher than having $R_{0}<R_{0_{\text{I-P,I-I}}}$. However, given that the underlying sub-epidemics in this case have $R_{0_{1}}=2$ and $R_{0_{2}}=1.66$ that are also bigger than the reference values, we show that the I-P and I-I methods reasonably capture the sub-epidemics using noisy data. And between these two, the I-P method is considered more robust for its nearly perfect $p_{\text{I-P}}\approx 1$ even at high σ=0.2. In comparison, the SM is significantly more sensitive to noise in terms of both low $p_{\text{SM}}$ and large variance $R_{0}$ distributions across all noise levels, as shown in figure 5c. This is expected as we showed theoretically in electronic supplementary material, section C(b), that the solutions to the SM system have a comparatively larger gradient with respect to its input observable statistics.

### 5.3. Estimation bias and error

By studying the $R_{0_{\text{I-P}}}$ distribution over D, we demonstrate under the I-P scheme the bias and error in estimating the overall reproduction number for $j_{S}$ against its two constituting sub-waves. We note that although the I-P method is used here for illustration, all three estimates for the reproduction number $R_{0_{\mu }}$ are qualitatively similar in response to varying $R_{0_{2}}$ and ${\bar{\tau }}_{d}$, as shown in electronic supplementary material, section D(c).

**Figure 6.**
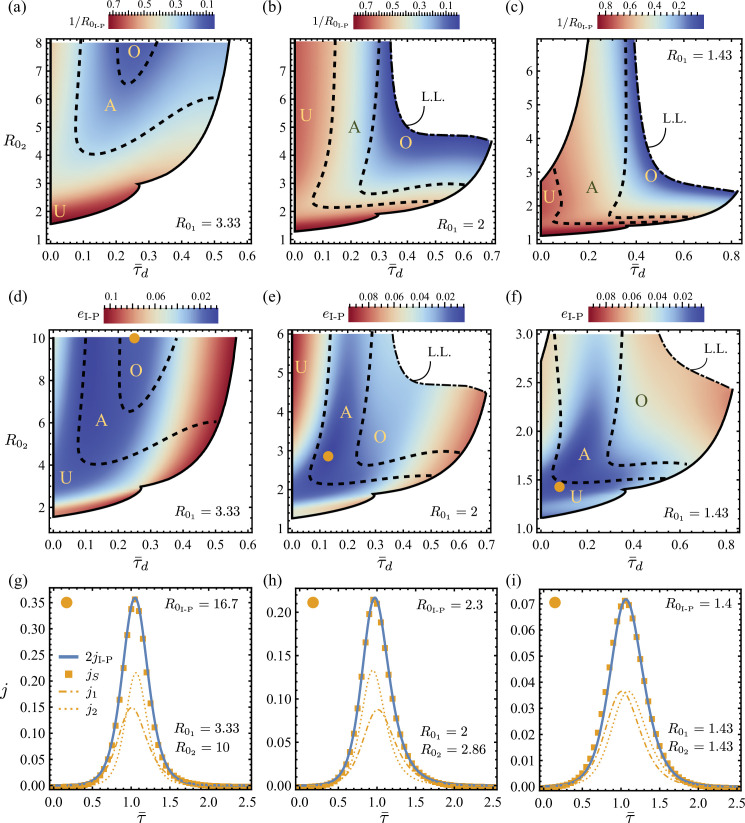
(a–c) Density plots for $R_{0_{\text{I-P}}}$ obtained for the aggregated incidence $j_{S}(\bar{\tau };{\bar{\tau }}_{d},R_{0_{1}},R_{0_{2}})$ using the I-P scheme. $k=1/R_{0_{\text{I-P}}}$ as a function of parameters ${\bar{\tau }}_{d}$ and $R_{0_{2}}$ is given for decreasing $R_{0_{1}}=3.3$, 2, 1.4 in (a–c). The inverse reproduction k is coloured for its boundedness 0<k<1. The solid black lines are boundaries for the unimodal domain D. We label as limit lines (L.L.), shown as dash–dotted curves, the boundaries where $R_{0_{\text{I-P}}}\to \infty$. The dashed lines separate D into labelled regions where $R_{0_{\text{I-P}}}$ underestimates (U), averages (A) or overestimates (O) the underlying dynamics $R_{0_{1}}$, $R_{0_{2}}$. (d–f) The $L^{2}$ error of the SIR estimation, $e_{\text{I-P}}$, plotted over the same parameters. (g–i) Simple SIR estimate for $j_{S}$ specified by the disc centre highlighted respectively in (d–f). Both the synthetic data (squares) and corresponding SIR model (solid lines) are given. $R_{0_{\text{I-P}}}$ is compared with $R_{0_{1}}$ and $R_{0_{1}}$, showing examples of overestimation, averaging and underestimation. We demonstrate that biases of both kinds can occur even though the estimated SIR theory for the aggregation appears to capture the data very well.

Figure 6a–c shows maps of D, where $R_{0_{\text{I-P}}}$ is computed for three different $R_{0_{1}}$. Depending on the comparison between $R_{0_{\text{I-P}}}$ against both of its underlying $R_{0_{1}}$ and $R_{0_{2}}$, three regions within D can be identified: (U) the $R_{0}$ underestimation region where $R_{0_{\text{I-P}}}$ is less than both $R_{0_{1,2}}$, (A) the $R_{0}$ averaged-estimation region where $R_{0_{\text{I-P}}}$ is in between $R_{0_{1}}$ and $R_{0_{2}}$ , and (O) the $R_{0}$ overestimation region where $R_{0_{\text{I-P}}}$ is greater than both $R_{0_{1}}$ and $R_{0_{2}}$. We see that if $R_{0_{2}}⪅R_{0_{1}}$, the I-P method always yields an $R_{0}$ estimate, $R_{0_{\text{I-P}}}$, which underestimates both of its underlying dynamics $R_{0_{1}}$ and $R_{0_{2}}$. Increasing the delay ${\bar{\tau }}_{d}$ in this case tends to generate smaller $R_{0_{\text{I-P}}}$ and thus exacerbates the $R_{0}$ underestimation. However, if $R_{0_{2}}>R_{0_{1}}$, there could be an initial interval of ${\bar{\tau }}_{d}$ for a given $R_{0_{2}}$ where $R_{0_{\text{I-P}}}$ increases and then decreases with ${\bar{\tau }}_{d}$. An example of this is discussed in electronic supplementary material, section D(b). That is for $R_{0_{2}}>R_{0_{1}}$, $R_{0_{\text{I-P}}}$ is generally no longer monotonic in ${\bar{\tau }}_{d}$ and instead can cross over regions from U to A and/or A to O. In other words, if the reproduction number of the second underlying wave is larger than that of the first, the delay can induce both underestimation or overestimation of the aggregated reproduction number. Conversely, if the reproduction number of the second wave is smaller than the first, the higher the delay between the underlying outbreaks, the higher the underestimation of the aggregated reproduction number.

Another metric that assesses the SIR estimation error is the $L^{2}$-distance between $j_{S}$ and $j_{\mu }$, defined as


(5.3) $$e_{\mu }={\left[\int_{0}^{\infty }{\left(j_{S}-j_{\mu }\right)}^{2}d\tau_{1}\right]}^{1/2}.$$

The distributions of $e_{\mu }$ for the I-P method, $e_{\text{I-P}}$, are plotted in figure 6d–f with the same $R_{0_{\text{I-P}}}$ boundaries separating regions U, A and O overlaid. We observe that areas of low $e_{\text{I-P}}$ can be found in all three regions, indicating little correlation between $e_{\text{I-P}}$ and the bias characteristics of the $R_{0}$ estimates discussed above. To illustrate this further, we show direct comparisons between $j_{S}$ and $j_{\text{I-P}}$ as functions of $\bar{\tau }$ in figure 6g–i for corresponding representative points labelled using solid discs in figure 6d–f. Although excellent overall agreement between the aggregated data and the SIR reconstruction is found in all three examples, the $R_{0}$ estimates are shown to differ from those of the underlying sub-outbreaks in all three possible ways, namely with an $R_{0}$ overestimation in figure 6g, a weighted average in figure 6h and an $R_{0}$ underestimation in figure 6i. This is also illustrated when comparing figure 6*a*–c and figure 6d–f: areas of low error, $e_{\text{I-P}}$, are found in all three regions, from overestimation to underestimation. Thus, it is difficult to determine, *a priori*, based on the aggregated incidence data alone, whether an SIR model is capable of recovering correctly the underlying reproduction number, and hence the epidemic dynamics. Furthermore, given a reasonable match between $j_{S}$ and $j_{\text{I-P}}$, we note that underestimation occurs when $R_{0}$ is close to unity. This is particularly alarming because this range is common for important and prevalent infectious diseases, such as influenza [27], tuberculosis [28] and Ebola [29], suggesting that their severity is more likely underestimated when data aggregation takes place.

On the other hand, if data aggregation is known or assumed, figure 6 could also be used to challenge the applicability of the simple SIR even if there is a good fit with the aggregated data. For example, having obtained a small error from estimation on the aggregated data, our results based on two sub-regional waves suggest that large values of $R_{0_{\text{I-P}}}$ (e.g. $R_{0_{\text{I-P}}}⪆5$) are expected to overestimate both of the contributing underlying epidemics (figure 6a), while small values of $R_{0_{\text{I-P}}}$ (e.g. $R_{0_{\text{I-P}}}⪅1.4$) tend to underestimate both underlying epidemics (figure 6d). Along the region of low error, $e_{\text{I-P}}$ (blue in figure 6d–f), $R_{0_{\text{I-P}}}$ generally increases in figure 6a–c. This could be used to estimate the bias in estimation of aggregated $R_{0}$: large values of $R_{0_{\text{I-P}}}$ are expected to overestimate both of the contributing sub-epidemics (figure 6a), while small values of $R_{0_{\text{I-P}}}$ tend to underestimate both underlying sub-epidemics (figure 6d).

## 6. Application to influenza

Finally, we analyse influenza data collected in the United States to illustrate the insights gained above. Influenza (flu) is a viral respiratory infectious disease that can cause mild to severe illness. It is estimated that in the United States, between 5 and 20% of the population are infected with influenza every year [27], resulting in between 12 000 and 61 000 deaths annually since 2010 [30]. Characterizing and monitoring the severity of influenza outbreaks are therefore critical for public health management. Note that the simple SIR model does not, of course, incorporate waning immunity, demography and viral evolution associated with influenza.

### 6.1. Onset optimized susceptible–infected–recovered estimates

To apply our SIR estimation algorithms, weekly incidence reports for the raw counts of individuals diagnosed with influenza-like illness (ILI) in the 2017−2018 and 2018−2019 seasons were accessed from the Centers for Disease Control and Prevention (CDC) [31]. More recent statistics after 2019 is not considered since it is masked by the COVID-19 pandemic. The time origin T=0 is set at the 40th week in the calendar year, according to CDC’s definition of the beginning of the flu year [32]. (Time variable T has unit of weeks.) The raw incidence counts for each state are normalized against the state’s population obtained from the US Census Bureau [33]. To account for ILI under-reporting [34,35] and generate normalized incident rates, ${\dot{J}}_{\text{flu}}(T),\;0\leq T\leq 51$, that are order-of-magnitude comparable to the national statistics [27], an amplification factor of one hundred is applied to the raw incidence counts. As a result, the $R_{0}$ estimates that we obtain in this study are plausible but not necessarily accurate with respect to the true influenza transmission dynamics during the considered periods. Importantly, implementing an artificial amplification does not compromise the consistency of the following results since the primary concern of the present study is to assess the biases of simple SIR estimation by comparing the performance of extraction methods applied on the same dataset.

Also, Krishnan *et al*. [22] discovered heterogeneity in the CDC influenza data associated with hierarchically aggregating sub-regional statistics. Particularly, disparities in the epidemic onset time were examined across different states. It was established that a shifted-SIR model that accommodates an onset delay D of up to 20 weeks in the onset of infection dynamics outperforms the standard SIR model in explaining the flu data. This onset delay is incorporated here by choosing an initial time $T_{0}\in \left\{0,1,...,D\right\}$ (in weeks) that minimizes the error in peak time prediction for the SM and one that optimizes the mean absolute error for the I-P method. Details of these optimized algorithms are found in electronic supplementary material, section E(a). The I-I method is excluded in our application to the flu data in this section due to the typical weekly data reporting of flu cases. Indeed, such reporting makes the data too coarse for confident identification of the inflexion point.

**Figure 7.**
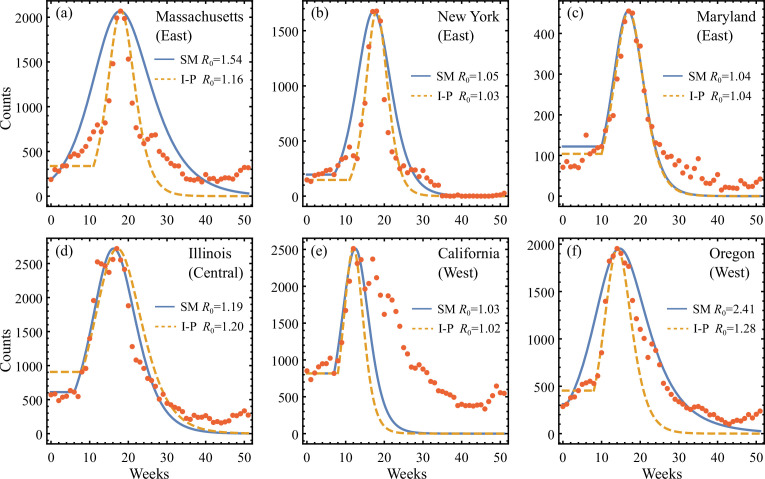
Application of the SIR estimation methods to 2017 influenza outbreaks in six US states. Comparison is made between the reported incidence counts in each state (circles) and the simple SIR $\dot{J}$ estimates given by the SM (solid line) and I-P method (dashed line). The optimized initial times used in (a–f) are *T*_0_ = 0, 7, 10, 6, 7, 0 for SM and *T*_0_ = 11,11, 10, 8, 8, 8 for I-P. Generally, the rising part of the data is shown to be well explained by the I-P method, while significant error can occur for the decaying part. This characteristic pattern is consistent with the findings of the two-waves model shown in figure 3.

Figure 7 shows the SIR estimations obtained for six representative states in 2017 using both the shooting and I-P methods. Overall, the optimized implementations ensure that both approaches capture the ${\dot{J}}_{\text{flu}}$ data at peak while the SM yields sensible agreement with data around its optimized initial time $T_{0}$, whereas the I-P system in general explains better the rising part of the incidence curves. A noticeable decrease in epidemic onset delay $T_{0}$ relative to the time origin is seen from the results of the I-P system moving east–west across the US from Massachusetts to California. This is consistent with the reporting for D studied by Krishnan *et al*. [22]. In comparison, $T_{0}$ that follows from the SM exhibits less geographic consistency, as a result of the particular choice of optimization objective. However, differences between the two methods diminish quickly when they adopt a similar $T_{0}$ value as given in figure 7c–e. And the corresponding $R_{0}$ estimates in these cases become nearly identical.

Furthermore, since by design, the present SIR estimates do not take into account the decay of ${\dot{J}}_{\text{flu}}$, the observed discrepancies in figure 7 between the post-peak measurements and predictions made by both methods show that using on-the-fly SIR estimates for post-peak epidemic predictions could lead to significant errors. This was also recently shown in the statistical analysis of Melikechi *et al*. [25]. For retrospective analyses, the presence of these errors clearly indicates the inadequacy of a simple SIR model. Additionally, we see that the post-peak errors found in the influenza data share similar characteristic features to those established for the synthetic two-epidemic model. For example, in figure 7d, the predicted decays are slower than the measurements, similar to the finding of the two-wave model with $R_{0_{1}}<R_{0_{2}}$. However, in figure 7e,f, the SIR estimates given by the I-P method drop markedly faster than the observed data, analogous to the synthetic case with $R_{0_{1}}>R_{0_{2}}$. The implications of these analogies are explored further next.

### 6.2. Data aggregation hypothesis

In the last section and associated figure 7 aiming to capture flu data with unimodal SIR models revealed patterns of error similar to those emerging from aiming to capture aggregated data emerging from underlying synthetic two-epidemic waves (discussed in §5.1 and figure 3). Here, we thus entertain the possibility that influenza data at state level may be constructed from aggregating data from sub-regions of distinct underlying epidemic dynamics. To this end, we introduce the single-SIR and double-SIR models that are fitted to the influenza data using nonlinear least-squares. Contrary to the single-SIR fit, the double-SIR fit uses the sum of two curves of incidence produced by SIR equations of equal population and initial condition η but different reproduction numbers $R_{0_{1}}$ and $R_{0_{2}}$. For both the single- and double-SIR fits, the generally non-zero onset shift $T_{s}$ (discussed in §6.1 and [22]) is an additional fitting parameter. Similarly, the relative delay $T_{d}$ between the two waves contributing to the double-SIR is also fitted. We give the system of ODEs for each fitted model in electronic supplementary material, section E(b).

**Figure 8.**
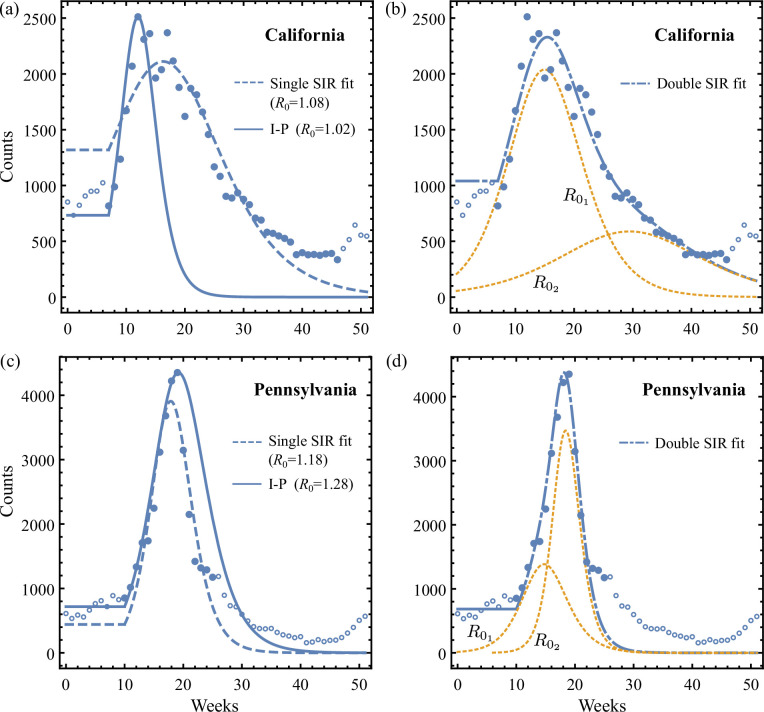
Comparison of the I-P method, fitted single- and double-SIR models applied to the 2017 influenza incidence data for California (a,b) and Pennsylvania (c,d). In all panels, the solid discs are data points used for nonlinear least-square fitting. The corresponding fit residuals are given in table 2. The open circles are points excluded, as explained in electronic supplementary material, section E(b). The dotted lines in (b,d) are the first and second sub-regional fitted SIR waves for the given state, whose sum defines the double-SIR model. The best-fit parameters are (a) β=2.17, $\eta =1.4\times 10^{-4}$, $R_{0}=1.08$, $T_{s}=11$; (b) γ=2.5, $\eta =2.8\times 10^{-5}$, $R_{0_{1}}=1.10$, $R_{0_{2}}=1.05$, $T_{s}=11$ and $T_{d}=0$; (c) β=3.03, $\eta =2.1\times 10^{-6}$, $R_{0}=1.18$, $T_{s}=4$; (d) γ=3.1, $\eta =1.4\times 10^{-5}$, $R_{0_{1}}=1.11$, $R_{0_{2}}=1.25$, $T_{s}=4$ and $T_{d}=10$. The double-SIR fit in both cases clearly outperforms single-SIR-based predictions. The use of a double SIR is informed by the test of the I-P method, revealing a match with the rising portion of the aggregated epidemic data curve, while a mismatch with the decreasing portion of the aggregated epidemic data curve.

Figure 8 compares the 2017 flu data in California and Pennsylvania against theories given by the onset-optimized I-P method, the fitted single-SIR model (figure 8a,c), as well as the double-SIR model (figure 8b,d). For the California case, the I-P method predicts a rapid decay of the outbreak that underestimates the real post-peak prevalence (figure 8a). The fitted single-SIR model produces better agreement for the decay, at the cost of not accurately capturing the initial epidemic rise. In comparison, the fitted double-SIR model (figure 8b) outperforms both previous estimates by more than 40% in terms of fit residuals, as tabulated in table 2. The best-fit values $R_{0_{1}}=1.10$, $R_{0_{2}}=1.05$ and $T_{d}=0$ are consistent with the synthetic two-wave model with $R_{0_{1}}<R_{0_{2}}$, where comparable post-peak error patterns are found when assessed by the I-P method. Therefore, it is reasonable to suspect that the California data consist of two, or possibly more, sub-epidemics of influenza with sufficiently distinct characteristics to result in distortion of the aggregate: introducing a second weaker wave than the first enables a slower decay of the summed system, relative to a single-SIR model, and thus yields an overall better match. This is reminiscent of the synthetic two-wave model in figure 3a–c, where a single-SIR model given by the I-P method has a faster decay than the synthetic aggregated data made of a relatively weaker trailing wave.

**Table 2.** Averaged absolute fit residuals (in counts per week) obtained by applying the I-P method; the fitted single- and double-SIR models with shifted onset time to the 2017 influenza incidence data in California and Pennsylvania. Comparisons are made for both the rising part of the data (small time range) and the overall data (large time range). The relative improvement is given between the fitted double SIR and the fitted single SIR.

	California		Pennsylvania	
data range	7≤T≤12	7≤T≤46	10≤T≤19	10≤T≤25
I-P method	180	733	165	671
fitted single SIR (electronic supplementary material, E.4)	365	187	336	327
fitted double SIR (electronic supplementary material, E.5)	237	115	190	242
relative improvement	35%	39%	43%	26%

Figure 8c shows the 2017 Pennsylvania case. Here, the I-P method overestimates the post-peak counts of incidence, whereas the fitted single-SIR model underestimates the epidemic peak. However, figure 8d shows that the rise and fall of the data are simultaneously captured by the double-SIR model, fitted using $R_{0_{1}}=1.11$, $R_{0_{2}}=1.25$ and $T_{d}=10$, gaining approximately 30% improvement in residual (table 2). Particularly, the observed change of slope during the incidence rises at around week 14 can be explained as the summed counts transitioning from being dominated by a weak leading wave to being dictated by a delayed second strong epidemic wave. Again here the observation that a single-SIR solution generated by the I-P method overestimates the post-peak incidence count suggests aggregation of multiple waves. In contrast, the fitted double-SIR model allows two waves to contribute. In the rising stage, both waves contribute to the pre-peak dynamics, while the post-peak incidence is dominated by the relatively stronger second epidemic wave ($R_{0_{2}}>R_{0_{1}}$). This composition is also seen in figure 3e,f, where for the I-P method to capture the rise of an aggregation, it overestimates the post-peak cases of the aggregated data.

In summary, we demonstrated that a single-SIR evolution proves insufficient in explaining the influenza incidence curve of a given state, and the error made by such a model may resemble those associated with data synthesized using a two-epidemic model. Specifically, if a single-wave theory matches the rising part of a unimodal measurement curve but consistently underestimates or overestimates its decaying part, existence of multiple sub-regional outbreaks within the state may be hypothesized: the rise and fall of an aggregation of incidence curves could be separately attributed to underlying waves of different strengths. We showed that in such cases fitting the data to a double-SIR model, for example, results in superior performance in terms of smaller residuals (table 2). This situation can be detected by examining the residuals of the pre- versus post-peak and comparing the I-P, with a single-SIR model fit. A clearly lower residual of the I-P compared to the single-SIR fit suggests the presence of double-SIR underlying dynamics (e.g. table 2).

### 6.3. Bias in $R_{0}$ estimation

**Figure 9.**
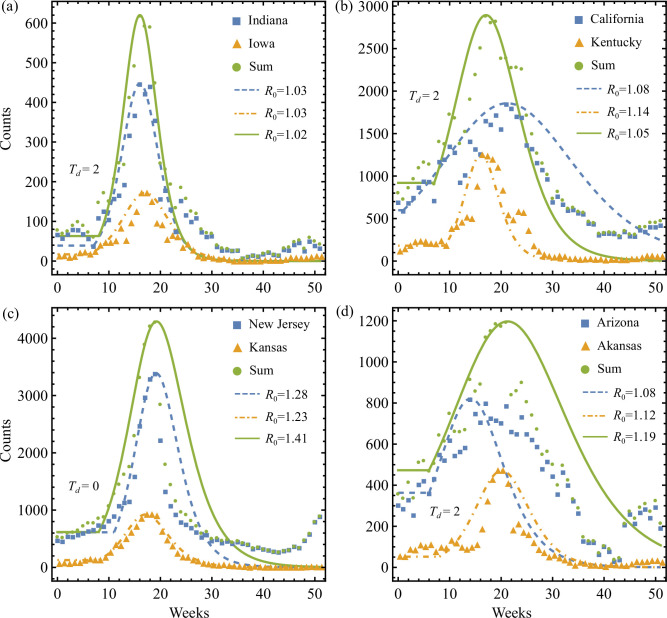
Examples of $R_{0}$ estimation bias due to aggregating influenza incidence counts from two different US states in 2017 (a,c) and 2018 (b,d). The relative delay between two states, $T_{d}$, is labelled in each case. For all panels, the I-P method is used for both state data (squares and triangles) and the summed counts (circles) to generate their simple SIR estimates given by the broken and solid lines, respectively. Both underestimation and overestimation of $R_{0}$ are possible. Here, although the $R_{0}$ bias appears small numerically, the corresponding $R_{0}$-based total infection predictions made by (6.1) can be significant, as seen in §6.3.


Next we study the effect of merging state data on the $R_{0}$ estimate obtained by the I-P method. Figure 9 shows examples of both $R_{0}$ underestimation and overestimation for aggregated influenza data. Figure 9a shows the Indiana and Iowa 2017 incidence counts and their sum, where the estimated $R_{0}=1.02$ for their sum underestimates its constituting epidemics that are comparable in onset time $T_{0}$ (with a relative delay of $T_{d}=2$) and have the same strength $R_{0}=1.03$. Underestimation is also found in figure 9b, where 2018 incidence data from California and Kentucky are aggregated. There the stronger Kentucky wave of $R_{0}=1.14$ lagged the California weak epidemic wave of $R_{0}=1.08$ by $T_{d}=2$, while their aggregation produces $R_{0}=1.05$, lower than both states. These two examples showcase the canonical configurations depicted in figure 1b,g, respectively. Although the relative differences between the $R_{0}$ estimates for component states and their aggregation in these two cases seem small, the severity of such $R_{0}$ underestimations cannot be overlooked when $R_{0}$ is used to approximate the cumulative number of infections, $J_{\infty }$, according to (4.7). Indeed, we evaluate $J_{\infty }$, under the η→0 assumption: we show that the relative difference in total incidence prediction between that of the aggregated curve and the sum of those due to the two underlying state curves is given by

(6.1) $$\begin{array}{lcr} & \Delta J=\frac{\left(N_{1}+N_{2})J_{\infty }(R_{0_{s}}\right)}{N_{1}J_{\infty }(R_{0_{1}})+N_{2}J_{\infty }(R_{0_{2}})}-1, & \end{array}$$

where $N_{1,2}$ are the total population of the two states, $R_{0_{1,2,s}}$ are the reproduction numbers estimated from the two states and the aggregated data, respectively. For the two cases given in figure 9a,b, ΔJ=−32% (i.e. difference of approximately 2000 incidences) and ΔJ=−39% (26 000 incidences), respectively. Thus, massive underestimation of $J_{\infty }$ prediction can result even from small bias in $R_{0}$ obtained from use of aggregated data biased by distinct epidemic sub-waves.

In contrast, summing the 2017 data for New Jersey and Kansas in figure 9c and the 2018 data for Arizona and Arkansas in figure 9d leads to a higher $R_{0}$ estimate for the aggregation compared to both of the underlying states in each case. Comparing figure 9a–c, the two aggregations are obtained using datasets of qualitatively similar wave shapes and relative onset delays, i.e. both analogous to the canonical case shown in figure 1*b*, but they give rise to opposite bias in $R_{0}$. We make the same observation when comparing figure 9b,d, where canonical configurations like figure 1g,h could also cause biased $R_{0}$ in both directions. Furthermore, using (6.1), the relative errors in $J_{\infty }$ estimate associated with the aggregations in figure 9c,d are, respectively, ΔJ=33% (15 000 incidences) and ΔJ=85% (14 000 incidences), implying a sizeable overestimation of cumulative incidence when $R_{0}$ is overestimated.

The examples in figure 9 illustrate how various $R_{0}$ estimation biases established for aggregated synthetic two sub-epidemics (e.g. figure 1) can be seen in real influenza data. These aggregation-induced $R_{0}$ estimation errors can lead to errors in the prediction of cumulative number of cases made from aggregated data that can be significant, translating into potentially costly intervention policies that either under- or over-prepare for epidemics across regions.

## 7. Surveillance prospect

So far we have discussed extensively how the SIR estimation methods developed in this work can be employed for retrospective analysis of epidemic data. Here we comment on the possible live surveillance and interpretation of pre-peak epidemic incidence data. We are particularly concerned with second sub-epidemics that are delayed in onset and relatively stronger (larger reproduction number) than the first sub-wave, yet both are still used for data aggregation. Indeed, when planning resource allocation and interventions using the early epidemic dynamics, one relies mostly on the first underlying weaker sub-epidemic wave. Hence, such planning is particularly ill-informed when aggregation of data involves the use of a second, delayed-onset sub-epidemic that is stronger than the first sub-epidemic. For perspective, we again turn to the synthetic two-wave aggregation discussed in §5.

**Figure 10.**
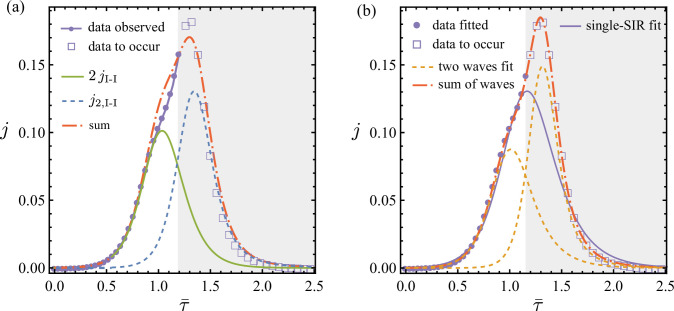
An example of monitoring aggregated epidemic data informed by two-step application of the I-I method. Continuous synthetic data $j_{S}$, which are the same as in figure 3f, are shown with purple dots and squares. In (a), the solid green line shows the same SIR estimation $2j_{\text{I-I}}$ as in figure 3f, i.e. applying the I-I method once. The dashed blue line shows the incidence curve inferred from applying the I-I method a second time, $j_{2,\text{I-I}}$, to the observed error Δj defined in (6.1). The dash–dotted red line gives the prediction $2j_{\text{I-I}}+j_{2,\text{I-I}}$ from the two-step I-I method. In (b), discrete data points (purple dots) are fitted using a double-SIR model (dash–dotted red line) and a single-SIR model (solid line). The two-wave fit recovers the parameters used in $j_{S}$ and the corresponding two sub-epidemics shown in dashed yellow lines. Only the data in the non-shaded areas of both (a) and (b) are used for the two-step I-I method and single- or double-SIR fits (mimicking a real-time prediction where the shaded area of the data is not known). We show in (b) that the single-SIR fit predicts poorly for the data to occur. But in both (a) and (b), a two-sub-epidemic hypothesis informed by the initial $j_{\text{I-I}}$ estimate correctly predicts the rise and decay of aggregated data in the shaded (blinded) area.

Particularly, considering the two underlying epidemic examples in figure 3f, we show that a delayed second epidemic can be detected as the epidemics and aggregation of data are ongoing and before reaching the apparent epidemic peak from the aggregation. For example, figure 10a,b shows data in non-shaded and shaded areas. In each case, only the data in the non-shaded area are used to infer the underlying dynamics. Figure 10a shows that the presence of a delayed second epidemic can be detected as the epidemic and data aggregation unfold. This can be seen for example when the I-I method is applied to the observed aggregated data $j_{S}$ generated by (3.3) after time $\bar{\tau }=0.86$ at the inflexion point of the aggregated data. The resulting SIR estimate $2j_{\text{I-I}}$ captures the data sufficiently well up to $\bar{\tau }\approx 1$, after which $j_{\text{I-I}}$ erroneously predicts decay in cases (green line). The presence of a bias introduced by second delayed-in-onset sub-epidemic wave can be detected with continuous monitoring of the error of prediction $\Delta j=j_{S}-2j_{\text{I-I}}$ (6.1) against the aggregated data. Indeed, Δj continues to increase with also a difference in the sign of the growth rates: by the time $\bar{\tau }=1.2$, an inflexion point in Δj is detectable. In this case, the I-I method can be used again to estimate a second wave $j_{2,\text{I-I}}$ that is added to update the overall prediction $2j_{\text{I-I}}+j_{2,\text{I-I}}$ (red line), i.e. resulting in a real-time two-step I-I method prediction. Similarly, once the growth in error is detected, and particularly with a shift in sign of rate of change of cases, a double-SIR fit introduced in §6.2 can be implemented as alternative to the two-step I-I method. This is shown in figure 10b, where the two sub-epidemic waves are also recovered well. The single-SIR fit obtained using observed data is also given in figure 10b. Its performance is satisfactory up to the very last time point, giving better accuracy than the first application of the I-I method shown in figure 10a. However, this result is potentially misleading and dangerous because the single-SIR fit misses the early signs of the delayed second wave that had been detected by the I-I method after the inflexion point. Consequently, we see in figure 10b that the single-SIR model considerably underestimates the epidemic data to occur, while a two-wave system, obtained either by the two-step I-I method in figure 10a or a least-square fit in figure 10b, correctly predicts the continued rise and the subsequent decay for the aggregated data.

## 8. Conclusions

We have systematically investigated the bias and error resulting from modelling aggregated epidemic data with the SIR theory. First, by inverting the analytical solution of the SIR equations, three distinct algebraic methods are formulated and solved to extract SIR parameters from a given epidemic incidence curve. Compared to a standard least-square fitting approach, where minimizing residual is the sole objective, the present methods aim to capture specific features of the incidence curve during different stages of an epidemic outbreak. Therefore, the existence and performance of SIR solutions that best match data around the initial and inflexion times (i.e. I-I method), or the initial and peak behaviours (i.e. shooting and I-P methods), can be directly assessed and compared. We also performed a sensitivity analysis of data noise to assess the applicability of these three methods given the expected data noise. We found that all three methods can deal with noise of reasonable sizes. The I-P and I-I methods are more likely to give SIR reconstructions for noisy data than the SM. The estimated SIR model, if established, carries a $R_{0}$ value that is most probably close to the reference value obtained for the unperturbed incidence curve by any given method.

To assess the issues arising from data aggregation, we generated synthetic two-sub-epidemic incidence curves by summing two independent SIR solutions of reproduction numbers $R_{0_{1}}$ and $R_{0_{2}}$, separated by a relative delay, $t_{d}$, between their onset times. We applied our three analytical SIR estimation methods to the synthetic aggregated incidence data and characterized the bias and error arising from the SIR estimations on the aggregated curves against the properties of the underlying sub-epidemics. Overall, we found that although in some cases the epidemic growth part can be captured well by our SIR estimates on the aggregated data, in other cases, very concerning prediction errors can occur for the peak or post-peak dynamics.

We also illustrated our approach of SIR estimation using the 2017 and 2018 US influenza data and showed that similar biases emerge from the real data compared to those of the synthetic two-sub-epidemic wave data analysis. Both underestimation and overestimation of the aggregated $R_{0}$ compared to the underlying reproduction numbers can occur when merging data from different states.

We hypothesized that given underlying sub-regional epidemics being sufficiently distinct in intensity or onset times, estimation bias of global reproduction number can emerge from data aggregation, for example, in large states or across states at the national level. This hypothesis is supported by the inadequate fit of single-mode SIR detection methods.

More precisely, modelling based on aggregated data when delay between the sub-epidemics and/or differences between the intensity of the sub-epidemics are present tends to significantly reduce the ability to capture the actual ground-truth epidemic dynamics. In particular, when aggregating sub-epidemic data, a number of insights emerge:

— For all offsets in onset time, $t_{d}$, between two underlying sub-epidemics when the second sub-epidemic is significantly weaker than the leading one, i.e. $R_{0_{1}}⪆R_{0_{2}}$, we find an underestimation of post-peak epidemic cases and an overall underestimation of the aggregated reproduction number compared to that of both underlying sub-epidemics. However, if $R_{0_{1}}<R_{0_{2}}$, the estimated SIR model exhibits different error patterns, and the corresponding aggregated $R_{0}$ could either be between $R_{0_{2}}$ and $R_{0_{1}}$ or can be an overestimation of both, depending on the delay in onset between the two sub-epidemics $t_{d}$.
— In an *a posteriori* analysis, excellent agreement between the aggregated data and the SIR estimation can emerge as measured by residual errors, despite a strong bias in the estimated aggregated $R_{0}$ when compared to the underlying sub-epidemic reproduction numbers. This is particularly concerning from a public health management perspective. Nonetheless, with prior knowledge or suspicion of heterogeneous data aggregation involving variable regional epidemic onsets and/or strengths, we find that for an aggregated $R_{0}$ close to unity (and no larger than ≈1.4), the underlying sub-epidemic severities are more likely *underestimated*, even in light of an overall low error in aggregated data capture. Conversely, *overestimation* of the aggregated $R_{0}$ is plausible when a large aggregated $R_{0}⪆5$ is found.
— One of the most misleading scenarios for real-time surveillance and planning of interventions/mitigation involves a second sub-epidemic that is stronger than the first, is delayed in onset, but yet is not sufficiently delayed to clearly induce a distinguishable bimodal aggregated curve. In such scenarios and with live epidemic surveillance in mind (prior to peak epidemic time), our framework can be used to detect the presence of such a second sub-epidemic. In this framework, the error estimation, (6.1), can be used for real-time update of the epidemic prediction to account for the second sub-epidemic, illustrated here with either a two-step I-I approach or a two-peak SIR model fit.

Overall, we find that for two epidemic waves, the stronger the trailing wave, the longer the temporal offset that maintains apparent erroneous unimodal aggregated data. In the special case of two equivalent epidemic strengths, however, the weaker the waves, the longer the offset that maintains apparent unimodal aggregated data.

## Data Availability

The paper has no new original data. The data used in our analysis were publicly available from CDC sources. These data were already freely available. The sources are listed as references in the paper [31,32]. Supplementary material is available online [36].
